# Small-Size Eight-Element MIMO Metamaterial Antenna with High Isolation Using Modal Significance Method

**DOI:** 10.3390/s24196266

**Published:** 2024-09-27

**Authors:** Tirado-Mendez Jose Alfredo, Jardon-Aguilar Hildeberto, Flores-Leal Ruben, Rangel-Merino Arturo, Perez-Miguel Angel, Gomez-Villanueva Ricardo

**Affiliations:** 1 Instituto Politécnico Nacional, Electrical Engineering Department, SEPI-ESIME-Zacatenco, Av. IPN S/N, Edif. 5., Ciudad de México 07300, Mexico; jtiradom@ipn.mx (T.-M.J.A.); arangelm@ipn.mx (R.-M.A.); 2 CINVESTAV-IPN, Telecommunications Section, Av. IPN 2508, San Pedro Zacatenco, Ciudad de México 07360, Mexico; rfleal@cinvestav.mx (F.-L.R.); aperezmi@cinvestav.mx (P.-M.A.); rgomez@cinvestav.mx (G.-V.R.)

**Keywords:** MIMO antenna, metamaterial, 8-elements, small size, octagonal SRR

## Abstract

This article presents a symmetrical reduced-size eight-element MIMO antenna array with high electromagnetic isolation among radiators. The array utilizes easy-to-build techniques to cover the n77 and n78 new radio (NR) bands. It is based on an octagonal double-negative metamaterial split-ring resonator (SRR), which enables a size reduction of over 50% for the radiators compared to a conventional disc monopole antenna by increasing the slow-wave factor. Additionally, due to the extreme proximity between the radiating elements in the array, the modal significance (*MS*) method was employed to identify which propagation modes had the most impact on the electromagnetic coupling among elements. This approach aimed to mitigate their effect by using an electromagnetic barrier, thereby enhancing electromagnetic isolation. The electromagnetic barriers, implemented with strip lines, achieved isolation values exceeding 20 dB for adjacent elements (<0.023 λ) and approaching 40 dB for opposite ones (<0.23 λ) after analyzing the surface current distribution by the *MS* method. The elements are arranged in axial symmetry, forming an octagon with each antenna port located on a side. The array occupies an area of 0.32 λ^2^ at 3.5 GHz, significantly smaller than previously published works. It exhibits excellent performance for MIMO applications, demonstrating an envelope correlation coefficient (*ECC*) below 0.0001, a total active reflection coefficient (*TARC*) lower than −10 dB for various incoming signals with random phases, and a diversity gain (*DG*) close to 20 dB.

## 1. Introduction

The development of new antenna topologies for 5G communications systems is experiencing significant growth. Many innovative structures are being proposed to meet the demanding requirements of modern systems, including the implementation of MIMO concepts to enhance channel capacity. For 5G MIMO applications operating below 6 GHz, a diverse range of antenna arrangements have been analyzed, ranging from two ports [1] to eight ports and beyond [2]. Each configuration presents unique advantages and complexities, with some prioritizing size reduction [3], others aiming to minimize cross-polarization levels [4], and still others focusing on reducing electromagnetic coupling between elements [4,5,6]. While the referenced configurations exhibit intriguing characteristics, they fall short of addressing the diverse requirements demanded by 5G applications.

For instance, although [1] demonstrates promising results in terms of multi-band operation, the antenna size for a two-port MIMO system is still considered substantial. Additionally, the presented TARC analysis is incomplete. This analysis should be self-comparative, varying the number of phases employed in the design, resulting in a more comprehensive assessment. Nevertheless, other works propose concepts that can be leveraged in future designs to enhance performance. In [2], a 16-antenna design is introduced, where the innovative aspect lies in the array configuration, placing each radiator in close proximity without significantly compromising performance. This isolation is largely achieved by the excitation method of each patch in the four-element subarrays, resulting in orthogonality and low electric field correlation.

Reference [3] presents a combination of fractals, the first based on a Sierpinsky carpet serving as the radiator array, and the second, a Hilbert curve, employed as a band-reject filter to enhance isolation between radiators. However, despite the reduced size, the usable bandwidth performance is limited when targeting authorized 5G bands. Consequently, the sole use of fractals is not a straightforward choice for wideband applications, although they offer multi-band capabilities.

These desired outcomes are achieved through a variety of methodologies, including the use of fractal geometries [7], electromagnetic band-gap structures [8], strategic element positioning and separation [9], metamaterials [10], and other approaches. Reference [9] highlights the strong correlation between the performance of a MIMO antenna array and the placement of its radiators and excitation points. Additionally, reference [10] provides evidence that metamaterials can enhance multiple performance aspects of an antenna. These materials allow for a reduction in radiator size without compromising efficiency, a significant advantage considering that smaller antennas typically suffer from decreased gain, bandwidth, and radiation efficiency. However, metamaterials mitigate many of these drawbacks, enabling the design of compact antennas without sacrificing overall performance.

MIMO antennas must accomplish different features, preserving the system bandwidth and the signal integrity by avoiding electromagnetic coupling among elements of the array. Most MIMO antenna developments are based on microstrip structures due to their easiness of integration, low profile, low cost, and low weight. 

Based on the aforementioned characteristics, this article proposes an eight-element low-profile MIMO antenna utilizing a double-negative metamaterial structure. The metamaterial structure takes the form of an array of octagonal embedded split-ring resonators (SRRs). By employing this structure as the antenna’s resonant element, it aims to achieve an increased slow-wave factor for the propagating electromagnetic wave, thereby reducing the overall antenna size compared to a conventional disc monopole. To mitigate electromagnetic coupling between the array elements, uncomplicated electromagnetic walls implemented with strip lines are introduced after analyzing the surface current distribution by employing the modal significance (*MS*) method.

The array composed of the eight elements is structured in an axial symmetry configuration, where each antenna port is located at the side of an octagon. The following sections present the antenna design and the array configuration, as well as simulated and measured results.

## 2. Performance Comparison of Disc Monopole, Circular SRR, and Octagonal SRR Antennas

This section presents a reduced-size MIMO antenna design utilizing a metamaterial SRR structure. The ring geometry is evolved from a circular silhouette to an octagonal shape, offering advantages over conventional disc monopoles and circular SRRs. For comparison, the proposed radiator is evaluated against both a disc monopole and a circular SRR antenna in terms of size and efficiency. The models were simulated on a RT/Duroid 5880 substrate with a dielectric permittivity of 2.2 and a thickness of 1.27 mm.

**Figure 1:**
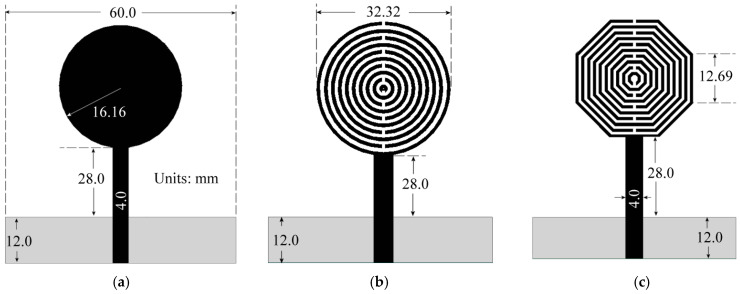
Comparison of: (**a**) conventional disc monopole, (**b**) circular SRR antenna, (**c**) octagonal SRR antenna.

According to Equation (1), a=F/1+2hπεrFlnπF/2h+1.77261/2, and Equation (2), $F=8.791\times 10^{9}⁄f_{r}\sqrt{\varepsilon_{r}}$, in [11], the radius, a, of the circumference for a disc monopole, working at 3.5 GHz, is around 16.16 mm, where h and $\varepsilon_{r}$*,* are the substrate thickness and permittivity. Now, taking into account the perimeter of the disc monopole of 101.53 mm, two metamaterial-based radiators were also designed, the first one using circular SRRs, whose outer ring has the same perimeter as the disc monopole, and the second antenna, using an octagonal SRR, having its outer ring perimeter equal to 101.53 mm, as depicted in Figure 1. In both cases, each ring is 0.2 mm wide and separated by 0.2 mm, too. 

**Figure 2:**
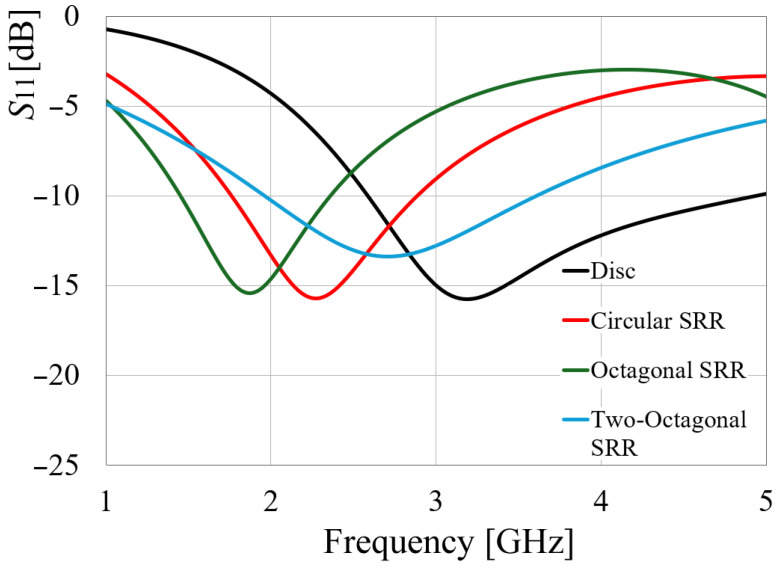
Simulated *S*_11_ parameter of conventional disc monopole, circular SRR, and octagonal SRR antennas.

**Figure 3:**
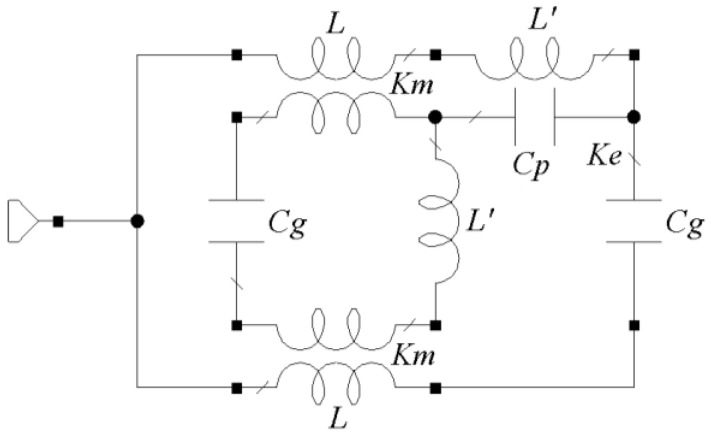
Equivalent circuit of a two embedded-octagonal SRR.

To complete the analysis of the effect of the resonant rings, the configuration shown in Figure 1c was modified to retain only the two larger rings. The frequency response of the four antennas is given in Figure 2. All antennas exhibit distinct resonant frequencies. The disc monopole resonates around 3.2 GHz, the circular SRR antenna at 2.27 GHz, and the octagonal SRR antenna at 1.86 GHz, while the two-octagonal-ring configuration resonates at 2.7 GHz, approximately. These findings align with expectations, as SRR integration into the structure increases the antenna’s slow-wave factor, resulting in an electric length exceeding that of a conventional element due to the enhanced reactive load induced by ring interaction. However, despite incorporating SRRs, the third antenna demonstrates a higher reactive load owing to the discontinuities present at each vertex, as detailed in Figure 3 [12]. 

In this model, *L* denotes the inductance associated with the individual line segments that constitute each ring. The value of this inductance is influenced by the presence of a parallel ring. The overall inductance is adjusted by the magnetic coupling factor *Km*. The capacitance generated at each ring gap is represented by *Cg*, and its value is influenced by the capacitance *Cp* between the rings through the electric coupling factor *Ke* [12]. An additional inductance, denoted by *L*′, accounts for the discontinuities introduced at the vertices of the octagonal shape. Then, the associated reactance given by *L*′ contributes to an increase in the total electric length of the structure compared to a circular ring. Following the procedure in [12], *L* = 3.9 nH, *L*′ = 0.9 nH, *Cp* 0.65 pF, *Cg* = 0.1 pF. Consequently, the resonance frequency of the octagonal SRR antenna is lower than that of the circular SRR radiator.

**Figure 4:**
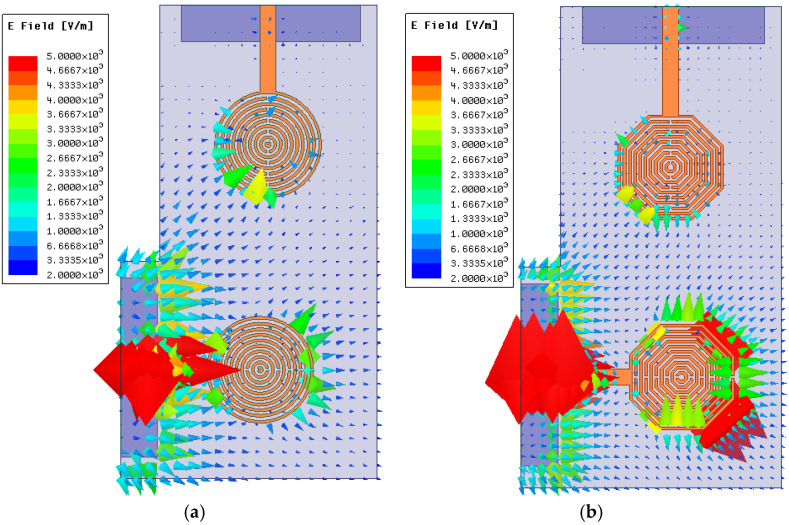
Comparison of the radiated electric field vector of: (**a**) circular SRR and (**b**) octagonal SRR antennas.

**Figure 5:**
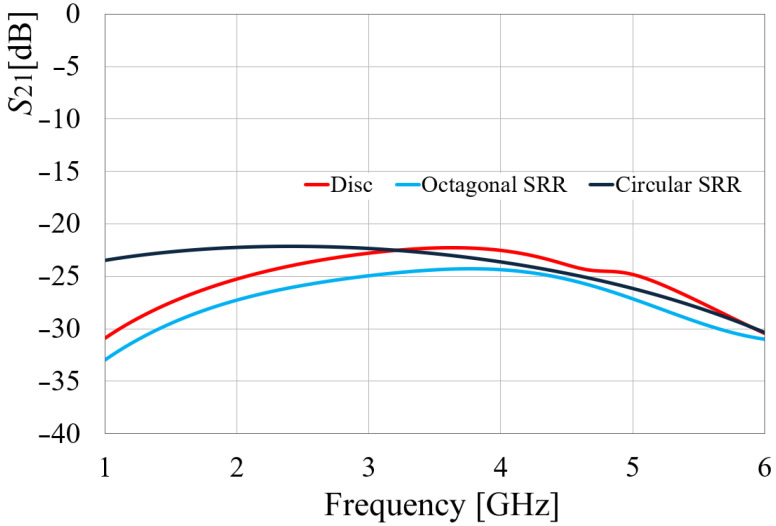
Simulated *S*_21_ response of the circular and octagonal SRR pair of radiators.

On the other hand, after carrying out an electromagnetic study using Ansys Electronics, the electric field radiation in the octagonal-shaped SRR antenna is more efficient compared to the circular SRR one. Also, for two closely positioned elements, electromagnetic isolation is also slightly better for the first geometry. Figure 4 depicts the electric field analysis of both configurations, and Figure 5 illustrates a comparison of the *S*_21_ parameter when two similar circular and octagonal SRR antennas are placed near each other.

In Figure 4, both antennas were excited with their respective resonant frequencies identified in Figure 2, with zero phase and a magnitude of 1 W, as well as the same scale to facilitate a fair comparison. The electromagnetic simulator was configured to represent the arrowhead size proportionally to the field line magnitude. These results demonstrate that the octagonal configuration exhibits higher radiation efficiency than the circular one. Additionally, the field coupled to the second antenna exhibits a larger magnitude in the circular SRR configuration. This finding can be further explained by analyzing the *S*_21_ response, as shown in Figure 5.

As displayed in Figure 5, the isolation in the octagonal SRR antennas is slightly better than in the circular SRR configuration, by almost 2 to 3 dB. Also, to the graphic, the *S*_21_ parameter of the configuration of two conventional disc monopoles was also added. In this case, as observed, the isolation at 3.5 GHz of the conventional disc monopoles is similar to the circular SRR configuration.

With these results, the octagonal SRR configuration presents a better performance concerning size reduction and electromagnetic isolation, since it accomplished a bigger reactive load, giving a larger electric length for a smaller ring size.

On the other hand, employing the “Antenna parameters” tool of the Ansys Electromagnetics, the resulting radiation efficiency of the radiator was 98%, approximately.

## 3. MIMO Antenna Design

**Figure 6:**
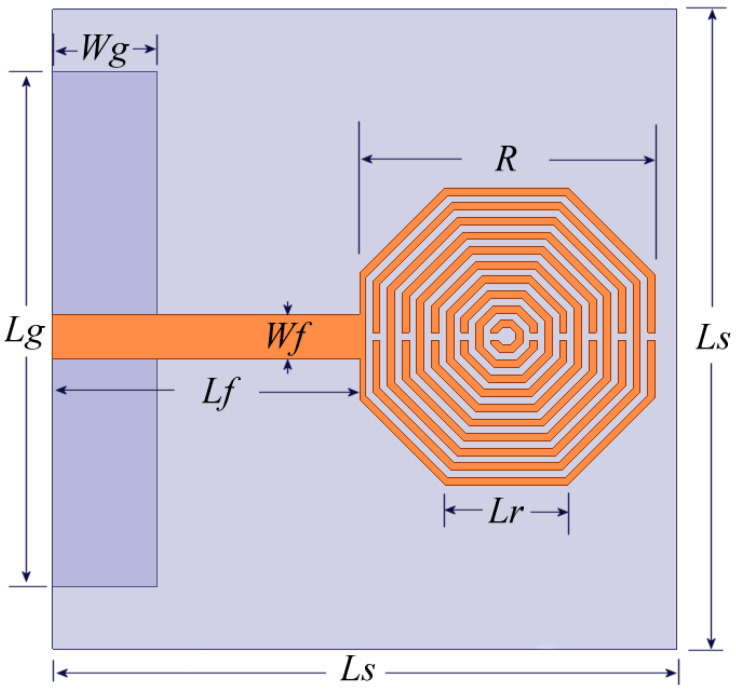
Proposal of antenna based on octagonal metamaterial SRR.

Considering the results given in Section 2, the antenna proposal based on a set of embedded metamaterial octagonal SRRs is based on the configuration given in Figure 1c. However, to shift the resonance to 3.5 GHz, a parametric analysis of the dimensions was executed by using Ansys Electronics, and the final configuration is depicted in Figure 6. As observed in this figure, the radiating element contains ten SRRs embedded in each other. The rings are 0.2 mm wide and separated by 0.2 mm, too. The gap inserted in each ring is set to 0.215 mm. *Ls* = 17 mm, *Lg* = 14 mm, *Wg* = 2.85 mm, *Lf* = 8.36 mm, *Wf* = 1.21 mm, *Lr* = 3.34 mm, and *R* = 8.03 mm. With these dimensions, it is clearly noted that the octagonal SRR antenna experiences a size reduction of more than 50% compared to a conventional disc monopole at the same resonant frequency.

**Figure 7:**
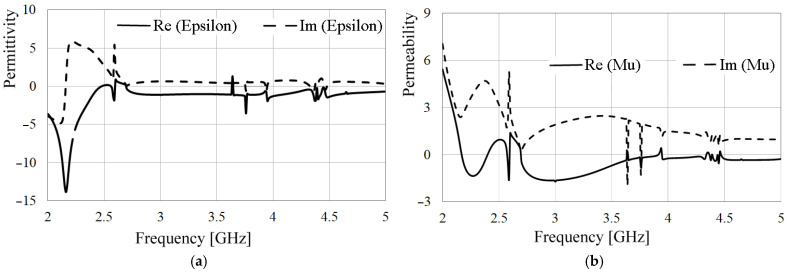
Extracted parameters of (**a**) Permittivity, (**b**) Permeability.

To ensure the metamaterial structure’s performance [13], the extracted permeability and permittivity are obtained following (1) to (4) and plotted in Figure 7. This procedure involved setting port number one on the edge of the feeding line and port number two in the radiation space above the SRRs.

Following [13], μ=nz and ε=n/z, where *n* is the refractive index. On the other hand, *z* is the impedance of the wave. The refractive index and the impedance are obtained as:

(1) $$z=\pm \sqrt{\frac{{\left(1+S_{11}\right)}^{2}-S_{21}^{2}}{{\left(1-S_{11}\right)}^{2}-S_{21}^{2}}}$$

(2) $$n=\pm \frac{1}{jL}\left(\frac{c}{\omega }\right){cosh}^{-1}\left(\frac{1-S_{11}^{2}+S_{21}^{2}}{2S_{21}}\right)$$

The signs in (1) and (2) are determined by:

(3) $$z^{\prime }\geq 0$$

(4) $$n^{\prime \prime }\geq 0$$

where (∙)′ and (∙)″ denote the real part and imaginary part operators, respectively.

**Figure 8:**
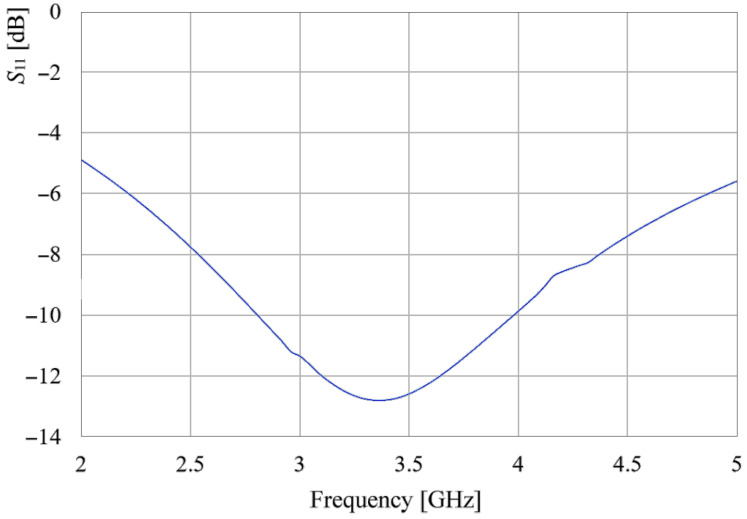
*S*_11_ parameter of the proposed metamaterial element.

Figure 7 shows the extracted real and imaginary components of the metamaterial structure’s permittivity and permeability, respectively. Particularly, for both components, the real part exhibits a negative response around the desired operating frequency, signifying a double-negative metamaterial. This behavior leads to a larger slow-wave factor on the structure compared to a conventional disc monopole, resulting in a resonant frequency of approximately 3.5 GHz with a significantly smaller radiator size. Figure 8 depicts the proposed antenna’s frequency response.

**Figure 9:**
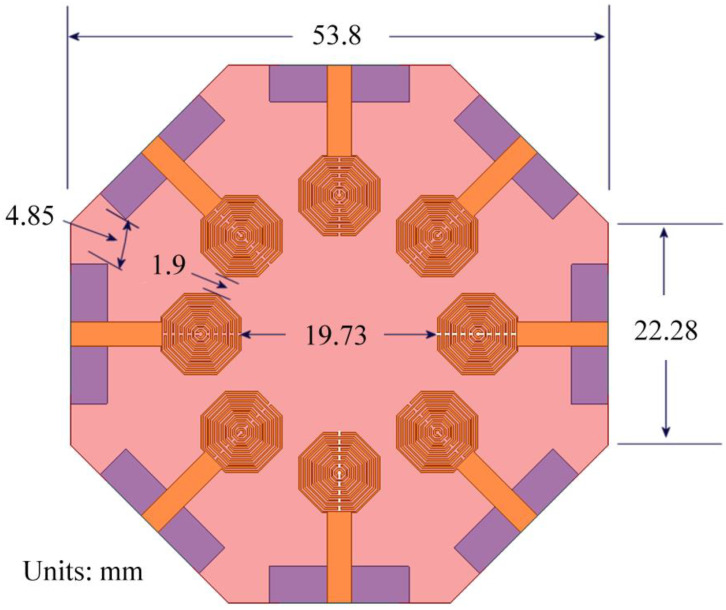
The 8-element metamaterial MIMO antenna proposal.

After corroborating that the antenna covers the 3.5 GHz band and has a port coupling below −10 dB, an 8-element with axial symmetry configuration is arranged in octagonal form as displayed in Figure 9.

From Figure 9, the area of the antenna is approximately 2400 mm^2^, close to 0.32 λ^2^ at 3.5 GHz, an exceedingly small size for an 8-port MIMO antenna, compared to previous published work, as will be seen below. The dimensions of each radiator were adjusted from those shown in Figure 6. 

**Figure 10:**
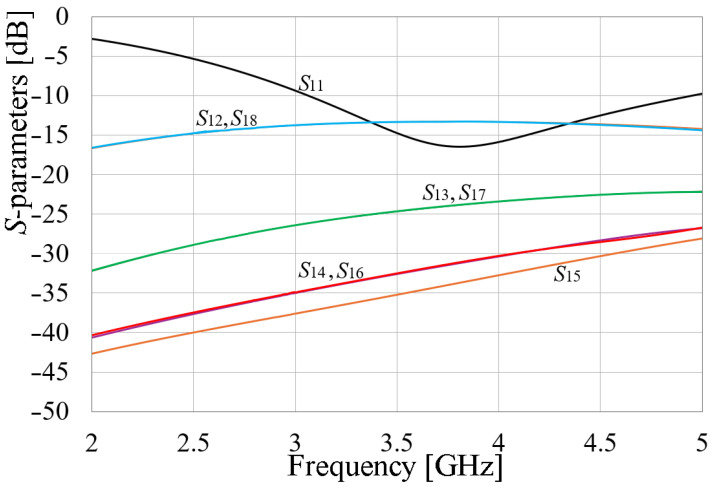
Simulated *S*-parameters of the 8-element MIMO metamaterial antenna.

As is known, the proximity of the radiators led to modifications in the electrical parameters. Consequently, new dimensions were obtained after another tuning process using Ansys Electronics. The resulting dimensions are *Lg* = 14 mm, *Wg* = 3.65 mm, *Lf* = 9 mm, *Wf* = 2.43 mm, *Lr* = 3.34 mm, and *R* = 8.03 mm. The tuning was focused on the ground plane and feeding line dimensions, while the remaining dimensions were preserved. These adjustments resulted in the simulated *S*-parameter values for the MIMO antenna shown in Figure 10.

In Figure 10, two important things must be considered: (a) because of the axial symmetry, all the *S*-parameters were related to port number 1, since other variations are the same. This means *S*_11_ = *S*_22_ = *S*_33_ = *S*_44_ = *S*_55_ = *S*_66_ = *S*_77_ = *S*_88_, as well as *S_ij_* = *S_ji_*, where *i* ≠ *j*, and *i* and *j* go from 1 to 8. Then, from this point, all results are given considering this simplification; (b) the isolation among elements is good except for those radiators which are the closest, for example, radiator one to radiator two and radiator one to radiator eight, achieving isolation values between 10 and 15 dB. This parameter is a particularly good result since the separation of the contiguous elements is close to 2 mm and the greatest distance between radiators is almost 20 mm, equivalent to 0.023*λ* and 0.23*λ*, respectively, being a smaller distance compared to other published works, as will be shown below. However, mutual isolation can be improved by using a passive structure without affecting the size and complexity of the MIMO array.

## 4. Mode Significance Analysis

Characteristic mode analysis (CMA) is a powerful technique for determining the dependence of propagated electromagnetic fields in a radiating structure [14]. The premise of this analysis is that the total current flowing through a structure excited by an electromagnetic wave can be expressed as a linear combination of *N*-orthogonal currents represented by their eigenvalues. These eigenvalues are dependent on the geometry and material of the radiator but not on the field that generates them.

**Figure 11:**
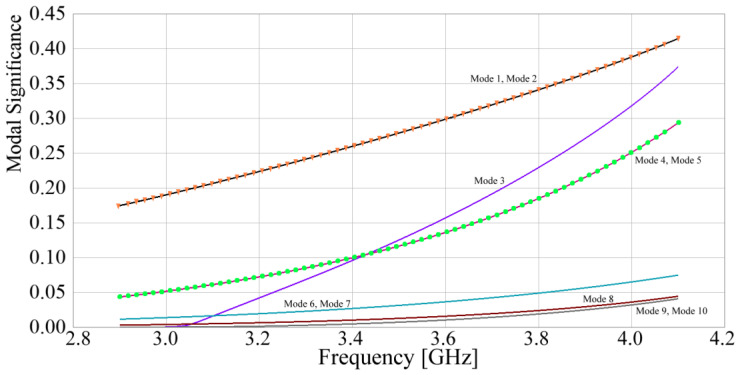
Modal significance of the structure shown in Figure 9.

A key concept within characteristic mode analysis is modal significance (*MS*), which determines the normalized amplitude of each modal current and, therefore, provides important information about the dominant mode in the radiation or propagation of the field on the structure in question. *MS* is given by:

(5) $$MS=\left|\frac{1}{1+j\lambda_{n}}\right|$$

where $\lambda_{n}$ is the eigenvalue of the *n*-th mode. Using the tool included in the CST Studio Suite 2024 simulation software, the *MS* of the structure shown in Figure 9 is determined, and the results are shown in Figure 11.

**Figure 12:**
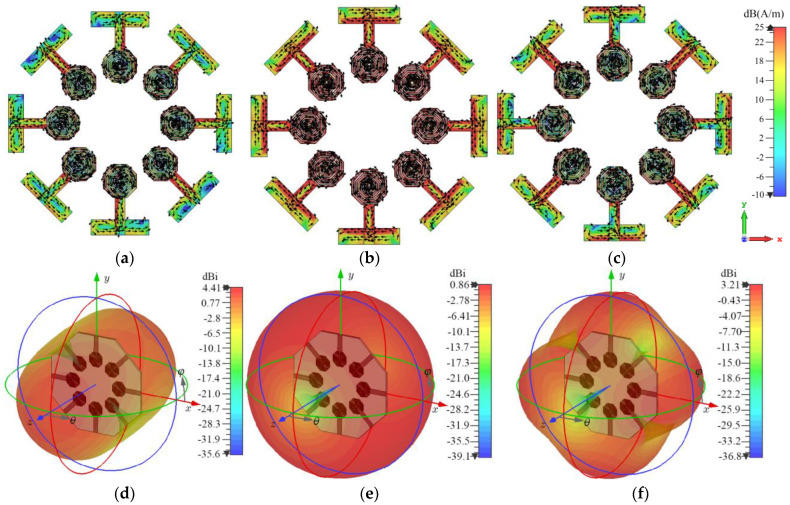
Current distribution for: (**a**) Mode 1, (**b**) Mode 3, and (**c**) Mode 4 and radiation pattern for: (**d**) Mode 1, (**e**) Mode 3, (**f**) Mode 4.

As observed in Figure 11, at the design frequency, modes 1 and 2 exhibit the greatest significance and are identical. These are followed in importance by modes 3, 4, and 5, where modes 4 and 5 also exhibit precisely the same behavior. Modes 6 through 10 have lower significance, by at least five times. In various antenna structures, dominant modes generated by a specific radiator tend to interact with other radiators through particular propagation paths between the elements. By analyzing these paths and employing barriers to block the propagation of these modes, electromagnetic isolation between radiators can be enhanced. Since the most significant modes, which produce the biggest effect, are isolated, in most cases, blocking other modes is not necessary. This allows for the design of compact electromagnetic structures with non-complex geometries. In this specific case, according to Figure 11, modes 1, 3, and 4 will be analyzed in greater detail to determine the electromagnetic interaction that is provoked among the array elements. Following this idea, Figure 12 shows the surface current distribution generated by the most significant modes 1, 3, and 4.

As observed in Figure 12, at 3.5 GHz for the three analyzed modes, the surface current intensity exhibits levels of up to 25 dBA/m on all radiators, denoted by a red color. This indicates a high level of electromagnetic induction among all elements. On the other hand, Figure 12d–f present the radiation pattern of the array for each significant mode.

As observed from the results presented in Figure 12, the most significant modes interact with all elements of the antenna array. Therefore, to minimize the electromagnetic interaction among radiators, metallic barriers are suggested to be employed. These barriers must cover all possible directions of propagation. This is unlike the work presented in [14], where the currents generated by the most significant modes did not interact with all radiators in the same manner and where DGS-based barriers were used that did not cover the entire ground plane. This is because the metal present in the areas where the barriers are not employed does not allow for significant propagation of the interfering modes. In contrast, the currents induced by each significant mode in this work generate fields that reach all radiators in the array.

**Figure 13:**
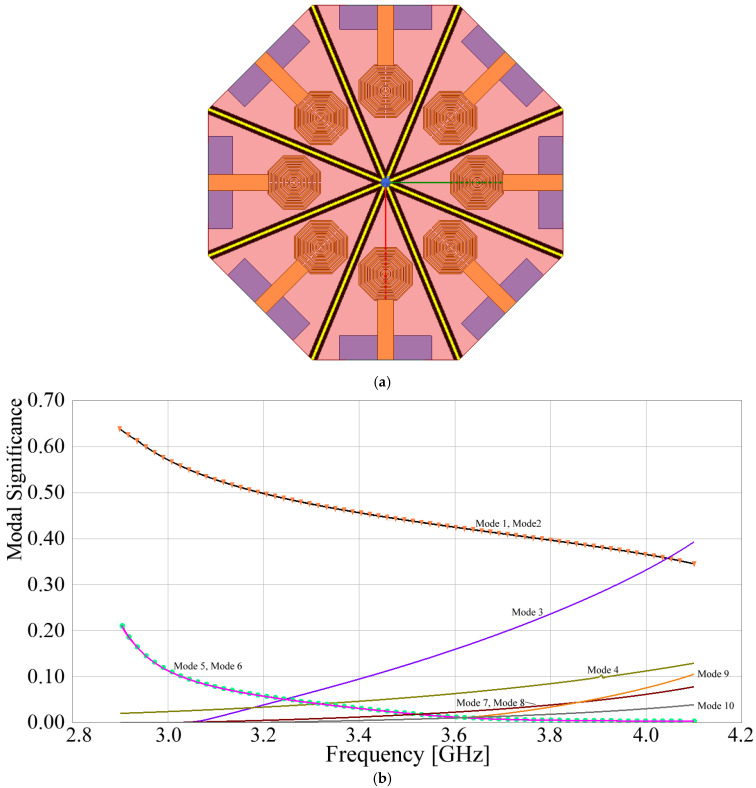
(**a**) Metamaterial MIMO antenna with electromagnetic walls, (**b**) Modal Significance.

To reduce the coupling levels among elements, an electromagnetic barrier implemented by metallic strips is proposed. Based on a similar behavior of metallic walls presented in [15], the structure used in this work most accomplish specific features: (a) size: since the radiators are closely spaced, the barrier must fit between the antennas, (b) isolations performance, (c) it does not deteriorate the main electrical and mechanical characteristics of the antenna, and (d) it must be easy to implement as a printed circuit. However, since the structure employed must be thin, it has to perform efficiently in order to isolate the most significant modes previously analyzed. Then, the proposed barrier structure is shown in Figure 13a.

In Figure 13a, the darkest lines are located on the bottom layer of the substrate, sharing the same plane as the ground planes. These lines have a width of 1.4 mm. The lightest lines, with a width of 0.4 mm, reside on the upper layer of the substrate, alongside the radiators. These dimensions were determined through a tuning process utilizing Ansys Electronics, with consideration given to the isolation level and port matching for each radiator.

**Figure 14:**
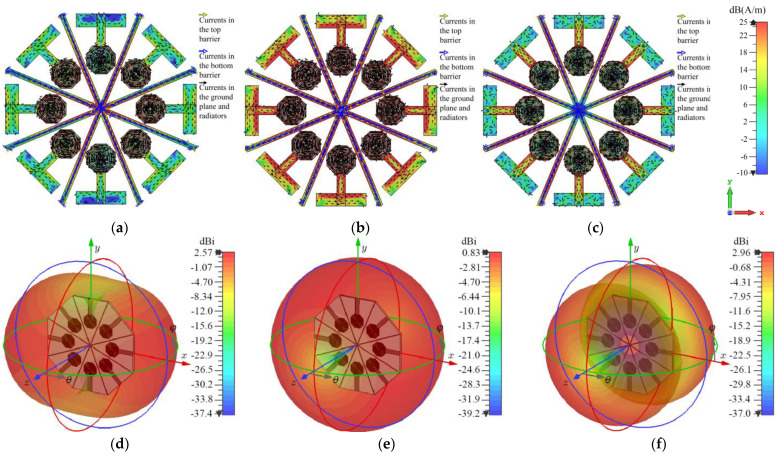
Current distribution with EM walls for: (**a**) Mode 1, (**b**) Mode 3, (**c**) Mode 4, and radiation patterns for: (**d**) Mode 1, (**e**) Mode 3, (**f**) Mode 4.

To probe the efficiency of the designed electromagnetic (EM) walls, the *MS* analysis was performed, but now over the structure presented in Figure 13a, showing the modal significance in Figure 13b, giving again the most significance to modes 1, 3, and 4. The resulting surface current distribution of these modes is depicted in Figure 14, as well as the radiation patterns for the most significant modes.

A comparison analysis of the surface current distribution on the radiators in Figure 12 and Figure 14 reveals a considerable difference between the configurations with and without electromagnetic barriers. The configuration that incorporates EM barriers exhibits a notably lower surface current intensity on the radiators compared to the configuration without these elements. This distinction is particularly evident in the feed lines and ground planes, where the intensity transitions from red hues to greenish-blue tones. This result suggests that the electromagnetic barriers effectively mitigate the induction of fields among the radiators.

**Figure 15:**
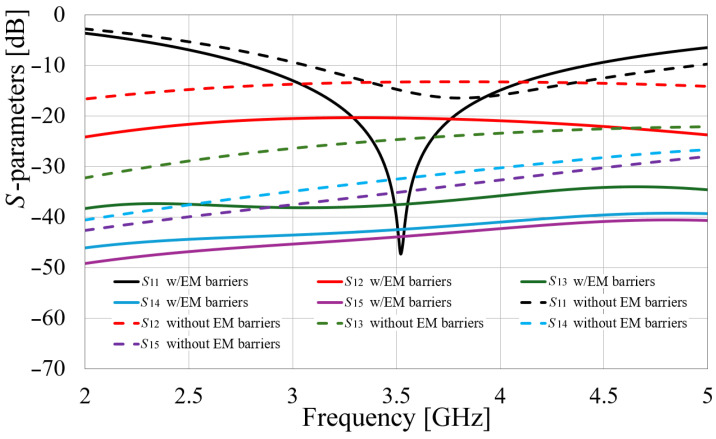
Comparison of simulated *S*-parameters of the MIMO antenna with and without EM barriers.

Once the array is optimized with the EM walls, the simulated *S*-parameter results are obtained and, taking advantage of the axial symmetry and for clarity, a comparison to the simulated *S*-parameters of the non-barrier configuration is given, considering the *S*_1*i*_ parameters for *i* = 1 to 5. This comparison is presented in Figure 15. This figure presents the improvement of the port matching, going from values below −15 dB to −45 dB, and increasing the electromagnetic isolation, from values about 15 dB to at least 20 dB all over the bandwidth, considering the adjacent radiators, and from 25 to 48 dB and higher isolation for non-adjacent radiators. This comparison is easily observed since the same-colored curves represent the same *S*-parameter, the solid ones for the configuration with barriers and the dotted ones belonging to the non-barrier configuration.

In summary, identifying the most significant modes enables the analysis of electromagnetic field inductions in the various radiators of the array and the proposal of an electromagnetic barrier to inhibit current paths between these elements. However, in this particular work, it was observed that all the most significant modes affected all radiators to some extent. Therefore, a barrier encompassing the entire inter-radiator section was required, preventing the limitation of these structures to strategically placed points of limited size, as proposed in [14]. Nevertheless, as shown in Figure 15, the use of these easily implemented barriers resulted in an increase in isolation.

**Figure 16:**
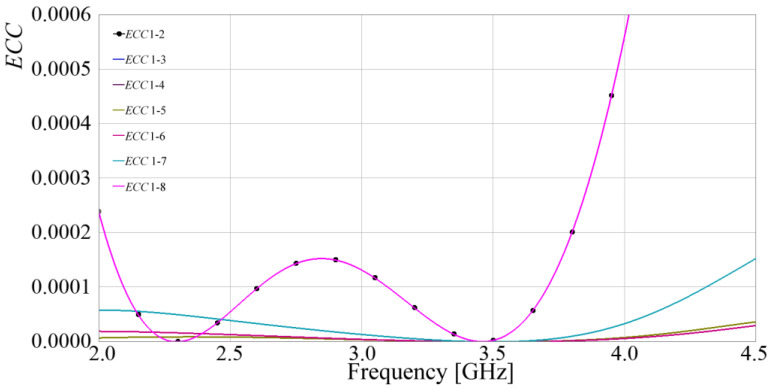
Simulated *ECC* between antenna 1 and the rest of the antennas.

Having achieved higher isolation, port matching, and bandwidth within a remarkably compact design, MIMO metrics were subsequently calculated. The simulated results are presented below. Equation (6) provides the mathematical expression for the envelope correlation coefficient (*ECC*) based on the S-parameters. Figure 16 illustrates the results, specifically the relationship between antenna 1 and the remaining radiators, taking into account that the other relationships are the same, due to the axial symmetry.

(6) $$ECC=\frac{{\left|S_{11}^{*}S_{12}+S_{21}^{*}S_{22}\right|}^{2}}{\left(1-{\left|S_{11}\right|}^{2}-{\left|S_{21}\right|}^{2}\right)\left(1-{\left|S_{22}\right|}^{2}-{\left|S_{12}\right|}^{2}\right)}$$

From Figure 16, it is observed that the *ECC* presents values much lower than 0.0002 at the desired frequency, showing the low E-field correlation among radiators, and as predicted, due to the axial symmetry, *ECC* 1 to 2 is equal to *ECC* 1 to 8, *ECC* 1 to 3 is equal to *ECC* 1 to 7, and *ECC* 1 to 4 is equal to *ECC* 1 to 6. 

**Figure 17:**
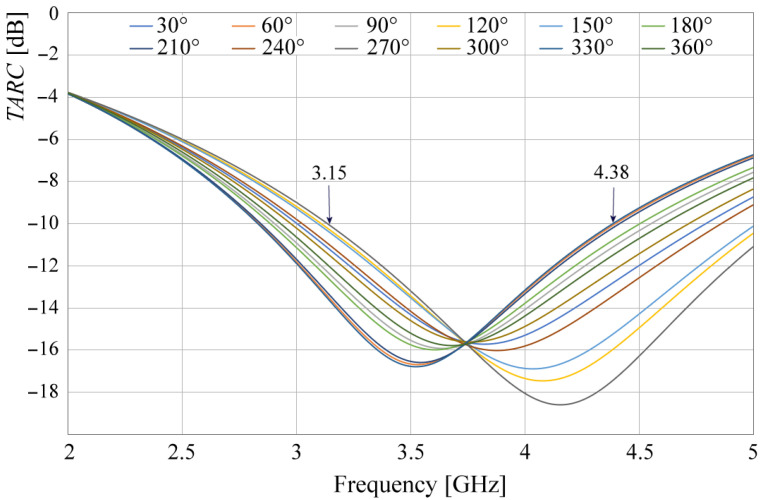
Simulated TARC for different $\theta_{0}$.

On the other hand, the other MIMO metric, the *TARC*, is given by (7).

(7) $$TARC=N^{-0.5}\sqrt{\sum_{i=1}^{N}{\left|\sum_{k=1}^{N}S_{ik}e^{{j\theta }_{k-1}}\right|}^{2}}$$

where *N* = 8. Since this equation involves the use of several phases ($\theta_{k}$), the number of curves could be enormous. Then, to facilitate the analysis and the understanding of results, phases $\theta_{1}$ to $\theta_{7}$ were set to 0° and $\theta_{0}$ was varied for different values. The results are presented in Figure 17.

The *TARC* plot in Figure 17 demonstrates the MIMO antenna’s excellent matching performance across the desired frequency range, with values below −10 dB from 3.15 to 4.38 GHz. This indicates that the antenna system can efficiently transmit and receive signals, even in the presence of random incoming signals from different directions. This feature is crucial for MIMO applications, as it ensures that each antenna element can operate independently without interfering with the others.

## 5. Construction and Measured Results

**Figure 18:**
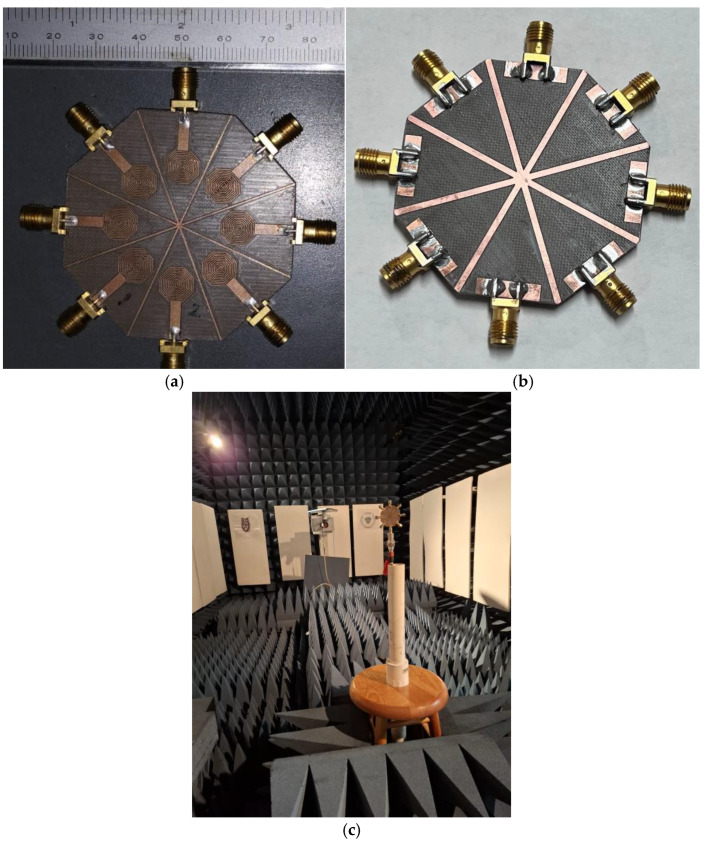
The 8-element MIMO antenna prototype, (**a**) front view, (**b**) back view, (**c**) inside the anechoic chamber.

**Figure 19:**
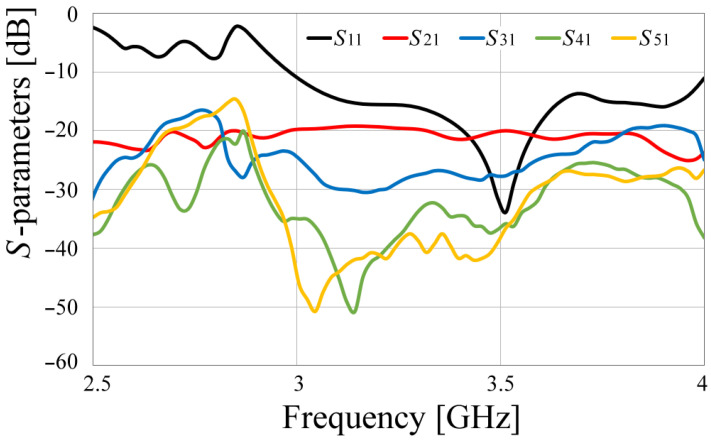
Measured *S*-parameters of the MIMO prototype antenna.

The configuration shown in Figure 13 is built, and the prototype is presented in Figure 18 and measured inside an anechoic chamber, shown in Figure 18c. The measured *S*-parameters of the array are given in Figure 19. Leveraging the axial symmetry configuration, for clarity in the results, the curves corresponding to the *S*_11_, *S*_21_, *S*_31_, *S*_41_, and *S*_51_ parameters were plotted, as another configuration presents similar results. In this figure, the resonance of the antenna at 3.5 GHz is shown, but the electromagnetic isolation among the antennas is also presented. As expected, the adjacent radiators to antenna number one, which was the element considered as the radiator of reference, possess the smallest isolation, achieving values slightly bigger than 20 dB, while the rest of the radiators show isolations close to 30 dB and around 40 dB when they are the opposite element.

**Figure 20:**
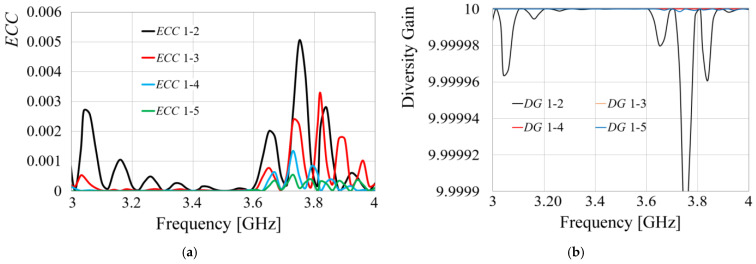
(**a**) Measured *ECC* of the prototype MIMO array, (**b**) Diversity Gain.

According to these results and considering (6) to calculate the *ECC* with the measurements, the outcomes regarding this figure of merit are presented in Figure 20a.

Figure 20a shows the results of the *ECC* between radiator 1 and the other radiators, as well as another combination due to axial symmetry. The *ECC* level at resonance is around 0.00002, which results in an exceptionally low E-field correlation among radiators in the array, a desirable property for MIMO applications, while Figure 20b presents the results obtained for the diversity gain, according to (8), showing values around 10. The resulting *DG* corresponds to 20 dB for all the relations of the antennas involved in the array, showing great performance for MIMO applications.

**Figure 21:**
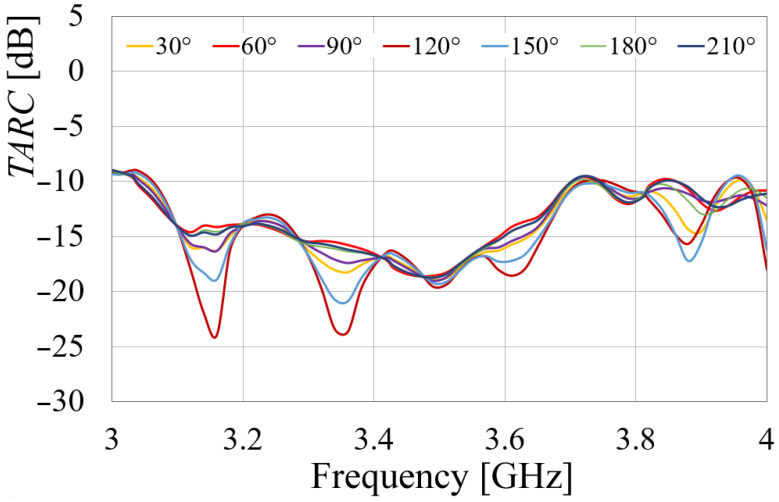
Measured *TARC* of the MIMO prototype.

Figure 21 displays the *TARC* calculated from measured results and applying Equation (7). Based on the graphics given in Figure 21, the system bandwidth was determined to be 3.06–3.71 GHz, following the same considerations as in Figure 17. This bandwidth achieves a *TARC* less than −10 dB, satisfying the requirements for MIMO applications.

(8) $$DG=10\sqrt{1-{\left(ECC\right)}^{2}}$$

**Figure 22:**
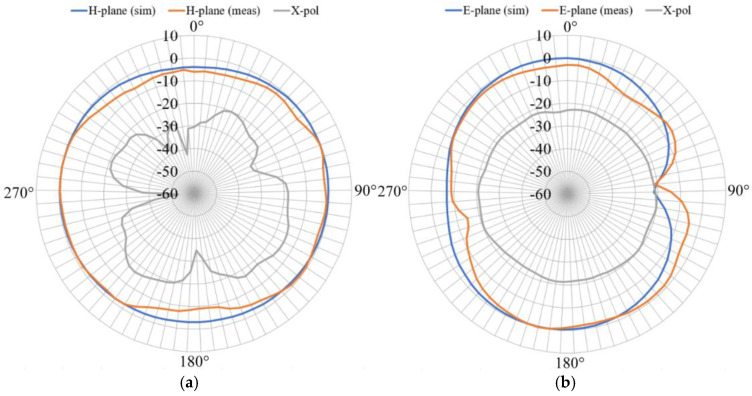
Normalized gain pattern. (**a**) H-plane, (**b**) E-plane.

Finally, the normalized gain pattern is presented in Figure 22, where the E- and H-plane are shown as well as the respective cross-polarization levels.

Figure 22 shows a comparison of the simulated and measured radiation patterns at the H- and E-planes, revealing excellent agreement. The measured cross-polarization at each plane is at least 10 dB lower than the corresponding co-polarization level. As anticipated, the H-plane pattern exhibits quasi-directional behavior, while the E-plane presents nulls at 90° and 270°, akin to a conventional monopole. However, the resulting gain is reduced compared to a monopole, reaching approximately −2 dBi, which represents the primary drawback associated with the antenna miniaturization.

Table 1 presents the main results concerning the radiation pattern of the array.

As expected in reduced-size antennas, the gain and directivity are reduced, since the real efficiency of small-size antennas is affected by lowering the gain, the radiation efficiency, and/or the bandwidth. In this case, the price to pay was the gain.

**Figure 23:**
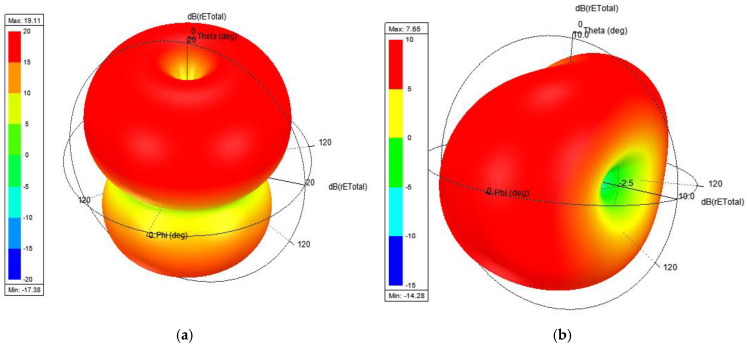
Simulated rE field, (**a**) total array, (**b**) independent radiator.

Figure 23 presents the simulated total rE field when all radiators are excited with the same phase and power.

As observed in Figure 23a, the array working at the same time, with equal phase and power, presents an omnidirectional pattern in the E-plane at theta equals 45°, while in the H-plane it shows nulls in theta equal to ±90°. Meanwhile, the radiation pattern independently presents a quasi-omnidirectional shape in the H-plane, while in the E-plane it shows some nulls at phi = 90° and 270°.

For comparison purposes, Table 2 shows the main characteristics of state-of-the-art publications related to eight-element MIMO antennas [15,16,17,18,19,20,21,22,23].

Table 2 clearly demonstrates that the proposed antenna boasts the smallest size compared to existing published works at the same operating frequency. For a fair comparison, the size is also compared regarding the wavelength. While working at similar frequencies, this work achieves superior *ECC* and *TARC*, maintaining comparable port isolation. Notably, the proposed design utilizes significantly less inter-radiator spacing than other structures, leading to enhanced MIMO array performance. Despite the reduced area, the isolation, *ECC*, and cross-polarization levels remain competitive with results presented in other referenced works.

It is important to note that many authors omit *TARC* results, a crucial parameter for characterizing the overall array performance, referencing the accepted and rejected energy when all MIMO antenna elements operate concurrently. While references [19,24] in Table 2 present a *TARC* curve, it is incompletely represented. The absence of a phase sweep hinders the ability to represent the random nature of incoming signals to the array [25].

## 6. Conclusions

This paper presents a highly compact eight-element metamaterial-based MIMO antenna. The design leverages octagonal embedded split-ring resonators (SRRs) as the radiating element, exceeding the performance of conventional circular SRR-based antennas. Compared to a disc monopole at 3.5 GHz, the proposed radiator boasts a size reduction exceeding 50%. Despite the exceptionally small inter-element spacing of less than 0.023λ, the proposed array exhibits superior isolation compared to existing works. The EM isolation was improved after employing the *MS* method to obtain the significance of the propagating modes and the effects on adjacent radiators. By using this method, an EM wall was implemented. Furthermore, the achieved *ECC*, *TARC*, and diversity gain comply with the stringent requirements of MIMO applications. The cross-polarization levels at both H- and E-planes are more than 20 dB lower than the co-polarization levels. Notably, the antenna design is straightforward and fabrication-friendly, devoid of the complex structures that pose challenges in other works, such as those referenced in Table 2. While the primary drawback of the structure is its reduced gain, this trade-off is inherent in designs prioritizing miniaturization.

## Figures and Tables

**Table 1:** Radiation field values at 3.5 GHz.

Parameter	Value
Peak Directivity	−2.0121 dB
Peak Gain	−1.98 dB
Peak System Gain	−1.67 dB
Radiation Efficiency	0.991

**Table 2:** Comparison of present work and state-of-the-art MIMO antennas.

Reference	Number of Ports	$\mathrm{Size}\;[{\mathrm{mm}}^{2}];\;\lambda_{0}^{2}$	Center Band [GHz]	Fractional BW [%]	*ECC*	*TARC* [dB]	Isolation [dB]
[16]	8	12,000; 0.83	2.5, 3.5, 4.9	42	NA	NA	≥10
[17]	8	11,250; 1.53	3.5, 4.9	25	≤0.1	NA	≥11.5
[18]	8	11,250; 1.53	3.5, 5	40	≤0.11	NA	≥15
[19]	8	11,275; 1.53	3.5, 4.7, 5.8	22	≤0.03	≤−20 ^2^	≥40
[20]	8	7737; 1.74	4.5, 7, 10	100 ^1^	≤0.001	NA	≥20
[21]	8	3600; 0.81	4.5	51	≤0.125	NA	≥15
[22]	8	13,689; 4.6	5.5	34	≤0.001	NA	≥20
[23]	6	3071; 0.78	4.8	27	≤0.029	≤−15	≥10
[24]	8	9800; 1.33	3.5, 5.5	18	≤0.03	≤−10 ^2^	≥20
**This work**	8	**2400; 0.32**	3.5	29	**≤0.00003**	≤−10	**≥20 to 40**

^1^ [20] 100% fractional BW with two notch bands. ^2^ [19,24] Incomplete *TARC* analysis.

## Data Availability

The original contributions presented in the study are included in the article, further inquiries can be directed to the corresponding author.
